# Adapting and pretesting the World Health Organization’s Caregiver Skills Training Program for children with autism and developmental disorders or delays in Hong Kong

**DOI:** 10.1038/s41598-022-21343-9

**Published:** 2022-10-08

**Authors:** Paul Wai-Ching Wong, Yan-Yin Lam, Janet Siu-Ping Lau, Hung-Kit Fok, The WHO CST Team, Chiara Servili, Erica Salomone, Laura Pacione, Stephanie Shire, Felicity Brown

**Affiliations:** 1 The Department of Social Work and Social Administration, The University of Hong Kong, Room 511, 5/F, The Jockey Club Tower, Centennial Campus, Pokfulam Road, Hong Kong, Special Administrative Region China; 2 The Faculty of Social Sciences, The University of Hong Kong, Hong Kong, Special Administrative Region China; 3 Department of Mental Health and Substance Use, World Health Organization, Geneva, Switzerland; 4 Department of Psychology, University of Milan-Bicocca, Milan, Italy; 5 Division of Child and Youth Mental Health, Department of Psychiatry, University of Toronto, Toronto, ON Canada; 6 Special Education and Clinical Sciences, College of Education, University of Oregon, Eugene, OR USA; 7 Research and Development Department, War Child Holland, Amsterdam, The Netherlands; 8 Amsterdam Institute of Social Science Research, University of Amsterdam, Amsterdam, The Netherlands

**Keywords:** Health services, Patient education, Psychology, Health care

## Abstract

The World Health Organization Caregiver Skills Training Program (WHO-CST) was developed to strengthen caregivers’ skills in supporting children with developmental delays and the caregivers’ well-being. The WHO-CST Hong Kong (HK) was adapted, and pre-pilot tested to support families with children suspected of having developmental delays and autism spectrum disorder and to empower the caregivers to foster their children’s learning, social communication, and adaptive behavior. A sequential mixed-methods research methodology was undertaken to examine the adaptation process and initial implementation experiences. The acceptability, feasibility, and perceived benefits of the WHO-CST were assessed using stakeholders’ and caregivers’ qualitative and caregivers’ quantitative pre- and post-intervention feedback. The data included materials generated from (1) three consultation meetings with stakeholders; (2) detailed reviews of the translated and adapted WHO-CST materials by master trainees (n = 10) trained by the WHO-CST representatives; (3) needs assessment focus group interviews with caregivers (n = 15) of children with autism spectrum disorder; and (4) pre- and post-CST program qualitative focus group interviews and quantitative evaluation. Consultation with stakeholders suggested that the program was acceptable for the local community, but the home visit and fidelity components were initially considered to be challenges towards the feasibility and sustainability of the program. Caregivers in the needs assessment focus groups gave widely diverse views about the program’s uniqueness, length, delivery mode, and the inclusion of videotaping in-home visits. Post-intervention comments by caregivers about the program were mainly positive, while the MTs were critical of the content and length of the training and fidelity process. As one of the first high-income locations to adopt the WHO-CST, the evaluation findings of the WHO-CST-HK indicate that it is feasible and acceptable to implement the program in a metropolitan area where families have busy work schedules and are very conscious of privacy issues. The study results suggest that the WHO-CST program in HK and other high-income countries require scaling up and further evaluation of its implementation in real community settings. This involves systemic and contextual changes to allow task-sharing between professionals and non-specialists at the macro level. Furthermore, technology should be used to support the supervision of non-specialists. In addition, easier access to the WHO-CST materials at the micro level is required to ensure equity, equality, diversity, and inclusion of diversified families of children with developmental delays.

## Introduction

Autism spectrum disorder (ASD) is characterized by the persistent display of at least two types of restricted behavior patterns, interests, or activities and a persistent deficit in social communication and interaction across multiple contexts^1^. The 2010 Global Burden of Disease study revealed that 52 million (1 in 132) people have ASD, and the World Health Organization (WHO) estimates the international prevalence of ASD is 0.76%^1^. Hong Kong (HK) has a population of 7.5 million; approximately 16% (n = 1,180,000) of people are aged under 20^2^. The number of children newly diagnosed with ASD has almost tripled, from 755 in 2006 to 2021 in 2015. ASD comprised over 60% of public hospital caseload for child and adolescent services in 2015–2016^3^.

The local government provides comprehensive support from early diagnosis and medical treatment to special education. The Child Assessment Centers support early identification and referral for further intensive services, and the Social Welfare Department and the Education Bureau provide special social and educational services^3^. Most families of children with childhood developmental issues receive services in the public system. The waiting time from speculation that a child has an ASD tendency to receiving assessment and diagnosis and then to receiving intensive services can take from 13 to 19.6 months^4^. Although this is within the timeline reported by other countries where diagnostic delays range from 7 to 30.6 months^5,6^, delayed access to early intervention raises serious equity issues, especially among those with low socioeconomic status, and significantly affects children’s health development. In addition, caregivers face the stressful demands of meeting both their own needs and the needs of the affected children, particularly during the waiting period when they have minimal support^7^.

## The World Health Organization Caregivers Skills Training Intervention (WHO-CST)

Previous studies have shown that parent-mediated programs play important roles in improving developmental outcomes for children with suspected ASD and developmental delays (DD). Caregivers’ stress can also be alleviated by encouraging caregivers to reach out for professional and informal support^8–10^. Hence, in consultation with experts and caregivers’ associations from all WHO regions and consideration of findings from several meta-analyses^11–13^, the WHO Caregiver Skills Training (WHO-CST) program was developed as a task-sharing parent-mediated program to support families of children with delays. The WHO-CST program is part of the Mental Health Gap Action Program (mhGAP) for scaling up care for mental, neurological, and substance use disorders in low- and middle-income countries (LMICs). The primary aim of the WHO-CST is for local specialists, referred to as master trainers (MT), to be trained by experts from WHO. The MTs then train non-specialist providers or facilitators, such as social workers, teachers, community leaders, and caregivers, to educate other caregivers of children aged from 2 to 9 with suspected ASD or DD. The WHO-CST consists of nine group sessions and three home visits, all building on the use of shared activities through play between caregivers and children during “home” and “play routines” as opportunities for promoting learning and development^14–16^. The trained facilitators then task-share the work with professionals and equip them with essential skills and knowledge to teach caregivers to engage their child in communication and play, promote adaptive behaviors and learning, and reduce challenging behavior^17^. To ensure conformity with evidence-based practices, the program requires both MTs and facilitators to complete training involving taught sessions and fidelity assessment. The fidelity assessment gauges competences in delivering the WHO-CST materials by rating video recordings of group sessions and home visits. The program has shown acceptability and feasibility in LMICs (e.g., Ethopia^17^, India^18^, Pakistan^16^) and high-income countries (HICs) (e.g., Italy^14^) and is currently undergoing field testing in over 30 countries.

It is important to note that (1) HK has a limited number of specialists in child and adolescent psychopathology in the public system, with great demand for free- to low-cost early assessment and intervention, (2) many families receive additional support from social services settings, and (3) there are cultural differences between Chinese and Western societies regarding values about play and the role of play in learning. Therefore, the WHO-CST has been culturally adapted and pre-piloted under the guidance of the WHO and Autism Speaks (AS) in Hong Kong since 2018. It has undergone a three-step sequential mixed-methods evaluation as suggested by the WHO.

The WHO proposes a three-step consecutive process to evaluate the feasibility, acceptability, and relevance of the WHO-CST program: (1) adaptation, (2) pre-pilot, and (3) pilot. In Phase 1 (the adaptation phase), the aim is to ensure cultural and contextual adaptation of the WHO-CST program before initial implementation. Phase 2 (the pre-pilot phase) assesses the program’s feasibility and acceptability when delivered by MTs and identifies requirements for further adaptations. Phase 3 (the pilot phase) uses a more rigorous research design and assesses the relevance and preliminary efficacy of the adapted WHO-CST program in the local setting when delivered by facilitators under the supervision of MTs. This paper reports on the first and second phases of the implementation process of the WHO-CST in HK conducted from May 2018 to July 2019. The main purpose of the research project was to introduce, culturally adapt, evaluate, and enhance the sustainability of an evidence-based, low-cost, task-sharing, parent-mediated program for caregivers of children with potential ASD or DD in HK.

## Methods

Details of the WHO-CST’s development, content, structure, and methodology have been described elsewhere^14–19^. The JC A-Connect Family Support team (hereinafter, the Team) closely followed the recommendations of the three-phase adaptation and evaluation by WHO: “Adaptation and Implementation Guide of the Parent Skills Training Program for Families with Children with Developmental Disorders and Delays”^20^ and “WHO Parent Skills Training Program for Caregivers of Children with Developmental Disorders: Monitoring and Evaluation Framework”^21^. In brief, Phase 1 focused on collecting stakeholders’ and caregivers’ opinions on adapting the WHO-CST to the HK context. Three adaptation meetings with local stakeholders, translation of the WHO-CST materials, and needs assessment focus groups with caregivers with service experiences were conducted concurrently. Phase 2 involved field testing of the adapted materials in a small-scale pre-pilot format to collect qualitative (i.e., group and individual) interviews and quantitative (i.e., uncontrolled) pre- and post-evaluation information to allow triangulation of data for analysis to inform the adaptability and feasibility of the WHO-CST-HK.

### Phase 1: Adaptation of the WHO-CST program

#### Translation of the WHO-CST materials

All English versions of printed materials (Field Test Version 1.01) were provided by WHO to the Team in May 2018. These included the introductory materials, Training of the Trainers Guide, caregivers’ booklet, facilitators’ booklet, and compulsory and suggested measures for research and publication guides. To facilitate maximum usage of the materials in other Chinese societies in the future, the materials were back-translated by two undergraduate students majoring in psychology and counseling, who were proficient in Cantonese, Mandarin, and English and strongly interested in ASD. Hence, an official written language instead of the Cantonese dialect was used. The Team reviewed the translated materials and finalized the draft for further reviews by the stakeholders in and between the three adaptation meetings. The draft version was made available for review by the local stakeholders with the goal of making the adapted WHO-CST suitable for implementation in Hong Kong and to be finalized after the third adaptation meeting for pilot testing in Phase 3.

#### Procedure and recruitment of local stakeholders and caregivers

From May 2018 to January 2019, potential stakeholders were introduced to the WHO-CST via site visits from the Team, which consisted of four MTs (i.e., two clinical psychologists and two counselors), a postdoctoral fellow (PDF), and a research assistant (RA) with a psychology background. After the medical and social services visits, interested individuals were invited (by email) to participate in the three adaptation meetings and review the translated WHO-CST materials. Thirty-four multi-sectorial stakeholders attended the meetings, including representatives from governmental agencies, community service providers, hospitals, non-governmental organizations (NGOs), funding agencies, university teachers and academics, and two WHO and AS representatives.

All meetings were conducted at the University of Hong Kong (HKU) and were audio- and videotaped for review and further data analysis. At the first adaptation meeting (November 2018), stakeholders were given the first version of the translated WHO-CST participant and facilitator booklets for initial review. Pre-set topics were discussed, including (1) sustainability; (2) training and supervision of MTs and facilitators; (3) adaptation and implementation plan; (4) data compiling and reporting; and (5) expected outcomes and deliverables. The second adaptation meeting was conducted in January 2019 and covered stakeholders’ feedback on the linguistic, cultural, contextual, and methodological considerations for the revised translated WHO-CST materials based on the feedback from the first meeting. The final adaptation meeting was held in January 2020 after the field testing of the adapted materials. The findings from the focus groups and field test were presented, and the stakeholders finalized and approved all materials printed in traditional Chinese.

From December 2018 to January 2019, NGOs were invited to refer eligible caregivers within their networks to participate in the needs assessment qualitative focus group interviews. Caregivers were eligible to join the interviews if they fulfilled the following criteria: (1) primary caregiver responsible for the role of parenting the child—this could be a biological parent (father or mother), guardian, or other adult family member; (2) a child aged 2–9 diagnosed with, or suspected of having ASD, developmental disorders or delays, as self-reported by the primary caregiver; (3) living together with the target child; and (4) able to communicate in Cantonese or English. Semi-structured focus groups were conducted with caregivers in December 2018 before the second adaptation meetings. These interviews explored (1) the participants’ needs and preferences concerning the parenting program; (2) possible barriers to attending the program and potential opportunities to link the program to other community services or support group activities; and (3) feasibility and acceptability of the WHO-CST content and strategies in HK. First, caregivers completed a short questionnaire about their sociodemographic characteristics, previous experiences participating in parent training programs, and their children’s clinical characteristics. They were then introduced to the aim, structure, and materials to be used during implementation of the adapted WHO-CST. They were invited to provide their opinions on prepared questions (see supplementary Table 1).

Focus groups, which lasted between 45 and 70 min, were led by the PDF and RA. They were audio recorded for data analysis. Fifteen caregivers (three Nepalese, twelve Chinese), all mothers, participated in the focus groups, one in group 1, two in group 2, and three in groups 3, 4, 5, and 6. Non-Chinese caregivers were invited to enhance the adaptability of the WHO-CST for the ethnic minorities population in HK. All focus group participants (age M = 38.1, SD = 2.2, range 34–41) identified themselves as their child’s primary caregiver. One was a working mother (part-time), and the rest were homemakers. Most caregivers (n = 14) had lived with the target child since birth. All had participated in various types of parent training programs, such as the Preschool Autism Communication Trial (PACT) (n = 2), Happy Parenting (n = 2), Louis Program (n = 2), The Hanen Program (n = 1), The SCERTS^®^ Model (n = 1), Applied Behavioral Analysis (n = 1), and a mindfulness program (n = 1) (see Table 1).

**Table 1:** Characteristics of caregiver-child dyads (n = 15) in the needs assessment focus groups.

Caregiver’s information								Child’s information				
Participant’s code	Nationality	Relationship with the child	Edu. level	Monthly household income (HKD)	Types of family	Number of children	Types of training program participated in their lifetime	Gender	Age	Edu. level	Diagnosis	Years of diagnosis
M01	Chinese	Mother	University	60,000 or above	Nuclear	2	None	Male	5	Kindergarten	ASD	2
M02	Chinese	Mother	Secondary	10,000–24,999	Nuclear	1	PACT, mindfulness, Happy Parenting	Male	8	Special School	ASD	5
M03	Chinese	Mother	University	60,000 or above	Nuclear	1	PACT, the Hanen Program, Happy Parenting	Male	6	Special School	ASD	3
M04	Chinese	Mother	University	60,000 or above	Nuclear	2	The SCERTS model	Male	7	Primary	ASD + ADHD	2
M05	Chinese	Mother	University	25,000–39,999	Nuclear	1	Louis Program	Male	3	Nursery	ASD	0.5
M06	Chinese	Mother	Secondary	10,000–24,999	Extended	2	Louis Program	Female	5	Special School	ASD + Genetic problems	1
M07	Chinese	Mother	University	60,000 or above	Nuclear	2	ABA, SEN certificate course	Male	7	Primary	ASD + language delay	3
M08	Chinese	Mother	University	25,000–39,999	Nuclear	1	Training courses from HHS/SAHK (not specified)	Male	7	Special School	ASD + ADHD	4
M09	Chinese	Mother	University	10,000–24,999	Nuclear	1	None	Male	5	Special School	ASD + ADHD	2
M10	Chinese	Mother	Secondary	10,000–24,999	Nuclear	1	Executive function	Male	7	Primary	ASD + ADHD	3
M11	Chinese	Mother	Secondary	25,000–39,999	Nuclear	2	Social class	Male	9	Primary	ASD + ADHD	6
M12	Chinese	Mother	University	10,000–24,999	Nuclear	2	Education talk related to language, social function	Male	8	Primary	ASD	4
M13	Nepalese	Mother	Secondary	40,000–59,999	Nuclear	2	Training courses from SAHK (not specified)	Female	5	Special School	ASD	4
M14	Nepalese	Mother	Secondary	10,000–24,999	Nuclear	2	Training courses from SAHK (not specified)	Male	5	Kindergarten	ASD + language delay	3
M15	Nepalese	Mother	University	25,000–39,999	Nuclear	2	None	Male	6	Special School	ASD + language delay	3

*ABD* applied behavioral analysis, *ADHD* attention deficient/hyperactive disorder, *ASD* autism spectrum disorder, *Edu*.  education, *HKD* Hong Kong Dollars, *HHS* Heep Hong Society, *PACT* Pre-school Autism Communication Trial, *SCERTS* Social Communication, Emotional Regulation, Transactional Support, *SEN* special education needs.

#### Training of MTs by the WHO-CST experts

Ten individuals (i.e., four from the Team and six from NGOs) with at least two years of clinical experience (M = 9.125, SD = 5.89, range 2–19) in counseling, clinical psychology, or social work were intensively trained at HKU for five days by two WHO-CST trainers in late January 2019 and supervised throughout the whole adaptation and evaluation process by material reviews and video coding discussions. Subsequently, MTs video recorded segments practicing the WHO-CST strategies with, a minimum of, two children with social and communication delays. The WHO-CST trainers reviewed and scored the recordings to evaluate each MT’s abilitiy to play with the children while demonstrating the skills to the caregivers, including setting up the environment, building and sustaining routines, promoting requests, supporting engagement, and promoting learning new skills.

#### Data analysis for Phase 1

The PDF and the RA summarized the suggestions from the three adaptation meetings. They read and re-read the summaries from all adaptation meetings before extracting major points from the five pre-set discussion topics. Audio records of the needs assessment focus groups with the 15 caregivers were transcribed verbatim in Cantonese by the RA under the supervision of the PDF. All interview transcripts were repeatedly read to become familiar with the data and make notes of early impressions. The PDF and the RA independently generated initial codes from the transcripts and iteratively agreed on a final code book. Data were coded using NVivo 11 software. All codes were then organized into initial themes and sub-themes until the PDF and RA reached an agreement with a reliability of at least 90%. The PDF, RA, and the first author met to review and modify the preliminary themes by assessing the chosen quotes associated with each theme. Finally, themes and sub-themes were refined, and rich data extracts illustrating each theme were selected. Conventional content analysis was used to detect manifest and latent meaning from the data^24,25^. Rigor was achieved through a process of reflexivity, documenting all analytic decisions, and leaving an audit trail. To ensure plausibility, the PDF and RA were not included in implementing the adapted WHO-CST program.

### Phase 2: Pre-pilot field test of the adapted materials

#### Recruitment of caregivers

From May to July 2019, all 10 MTs were involved in conducting an uncontrolled pre-pilot with caregivers recruited through the NGOs. They assessed the preliminary feasibility of delivering the WHO-CST-HK in its current format and identified potentially significant issues that required redressal before training facilitators in the pilot phase. Convenience sampling was adopted, and email invitations and flyers were sent to collaborating NGOs to be disseminated to their service users’ databases and social networks. Caregivers who met the following inclusion criteria were eligible to participate: (1) at least 18 years old; (2) responsible for the role of parenting the child; (3) living with the target child; (4) able to communicate in Cantonese; and (5) accessible by phone and planning to stay in Hong Kong for at least six months. Caregivers were excluded if they had a physical or mental condition that required hospitalization or frequent outpatient visits (over twice a month).

In addition, the target children were required to satisfy the following inclusion criteria: (1) 2–9 years old; and (2) screened positive on any of the modified ten questions screen questionnaire items for developmental delay and disorders, which assess physical ability, and ability to learn and speak^23^. This screening questionnaire was developed as a rapid and low-cost diagnostic tool for professionals to identify children with severe developmental disabilities in areas where resources are scarce^16^. Its reliability was tested internationally and was validated with Cronbach’s alpha falling within the range of 0.6–0.8 within the population of children between 2 and 9 years old^22^. Two of the items focused on physical disabilities (blindness and deafness), which were in the exclusion criteria for this study since including children with physical disabilities would complicate the implementation of the CST as recommended by the WHO. Therefore, the two items were removed, resulting in the questionnaire's modified version. Children were excluded if they had comorbid physical and mental conditions such that the child was currently hospitalized. Characteristics of caregivers and children who participated in the intervention are reported in Table 2.

**Table 2:** Demographics of caregivers participated in phase two (n = 42).

	n (%)	Mean (S.D.)
**Primary caregiver**		
Female	33 (78.6%)	
Age		37.59 (4.84)
**Education level of the participants**		
Primary	2 (4.8%)	
Secondary	16 (38.1%)	
Post-secondary	10 (23.8%)	
University or above	14 (33.3%)	
**Place of birth of the participants**		
Hong Kong	25 (59.5%)	
Mainland	17 (40.5%)	
**Marital status**		
Single	2 (4.8%)	
Married	39 (92.9%)	
Divorce	1 (2.4%)	
**Occupation**		
Work outside	23 (57.1%)	
Work at home	7 (16.7%)	
Unemployed	11 (26.2%)	
**Targeted child**		
Male	30 (71.4%)	
Chronological age (years)		3.91 (1.05)
Age at diagnosis (years)		2.79 (1.12)
**Sibling**		
With developmental delay	9 (21.4%)	
Without developmental delay	18 (42.9%)	
No sibling	14 (33.3%)	
Autism severity (Modified Ten Questions Screen)		1.81 (1.2)
Service*		
School support	15 (35.7%)	
Behavioural therapy	20 (47.6%)	
Speech therapy	14 (33.3%)	
Suggestion from traditional medicine therapist	9 (21.4%)	
Traditional/ assistive therapy	6 (14.3%)	
Information on services	3 (7.14%)	
Information about child’s issue	7 (16.7%)	
Information on parenting	5 (11.9%)	
Others	2 (4.67%)	
No support	9 (21.4%)	

*Child maybe receiving multiple services simultaneously.

#### Procedure

A briefing session was conducted at HKU, in partnership with NGOs, to recruit caregivers to join the pre-pilot trial. The pre-pilot WHO-CST-HK was delivered by a pair of MTs for each group. One MT was mainly responsible for the delivery of the WHO-CST, while the other MT acted as an assistant and observer to record questions or comments raised by the caregivers that could indicate improvement in the program’s comprehensibility, acceptability, and relevance. Feedback from MTs and caregivers was collected throughout the pre-pilot using an online survey tool accessible on hand-held devices, such as smartphones or tablets, immediately after each session. A RA would remind those who could not stay after the sessions to fill in the surveys if the system indicated that they had not done so. No incentives were given to the caregivers for research participation. In accordance with the WHO-CST research guidelines, the full delivery of the intervention was video recorded for review when necessary.

#### Data collection and measures

##### Qualitative assessment of feasibility and acceptability

**Figure 1:**
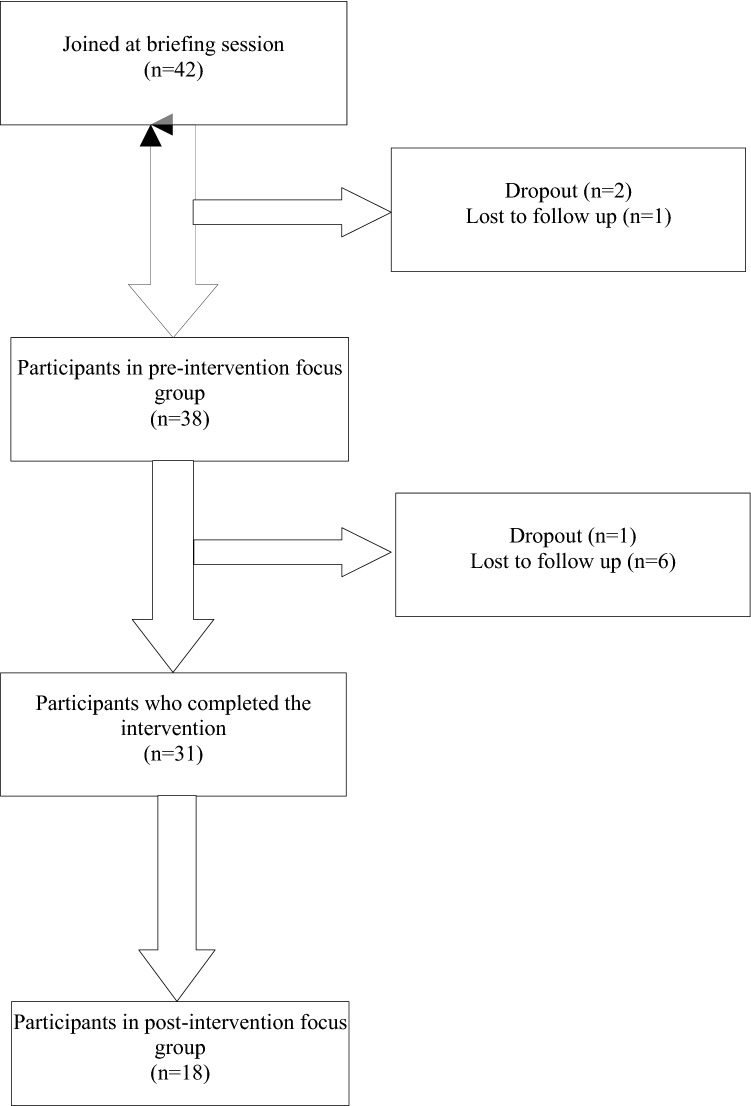
Phase 2 sample size at different time points.

Pre- and post-intervention focus groups were conducted with caregivers. During both focus groups, guides developed by WHO were used to direct the flow of the focus group discussion (see Supplementary Tables 1.2 and 1.3, respectively). The Chinese version of the guides was adapted by the PDF and RA and circulated and agreed upon among the Team. The PDF and RA conducted all focus groups. All participants gave full written consent. Forty-two caregivers of children with ASD initially joined this stage of the study. Approximately 80% of the caregivers were female (age M = 37.12, SD = 4.16, range 30–44), 21.4% were male (age M = 39.44, SD = 6.82, range 30–49) (see Table 2). Among these caregivers, 38 joined pre-intervention focus groups. Thirty-one caregivers attended the WHO-CST pre-pilot sessions, but some terminated due to scheduling issues or were lost in the follow-up, resulting in 18 caregivers remaining to participate in post-intervention focus groups (see Fig. 1).

One-to-one interviews with MTs were conducted before and after the pre-pilot (see supplementary Table 1.4). The interviews explored the MTs’ opinions, experiences, and suggestions for improvement in delivering the intervention and supporting facilitators. The PDF and RA led the interviews, and all MTs participated in the pre- and post-intervention individual interviews.

##### Quantitative assessment of feasibility and acceptability

All participating caregivers were required to complete sets of specific compulsory and recommended measures suggested by WHO-CST experts and chosen by the Team. The quantitative data were collected before, during, and immediately after the pre-pilot.

##### Quantitative assessment of feasibility

*Attendance tracking *Attendance was tracked by the MTs and researcher for both group and home visit sessions.

*Adherence to home practices *A caregiver diary was used to evaluate the caregivers’ experiences using the strategies they learned during class sessions in their home practices. They reported their confidence level, comfort in using the strategies, and how difficult, effortful, and natural the caregiver perceived the strategies to be^28^. Each caregiver was invited to complete the diary in accordance with their experience. This instrument was used at post-session 4 (mid-WHO-CST program) and during the third home visit (after the completion of the WHO-CST program).

##### Quantitative assessment of acceptability

All interested caregivers from six pre-pilot groups were asked to complete a set of 20 questionnaires throughout their participation in Phase 2. Questionnaires included one screening tool; one pre-intervention and one post-intervention questionnaire, which asked for demographic and service history information during baseline measurement; eight caregivers’ feedback on CST sessions; three knowledge and skills tests; three quality of life tests; and three caregivers’ feedback on home visits.

*Post-session feedback from caregivers *Each caregiver completed the post-session caregiver feedback form. These forms enabled participants to share their opinions about the quality, usefulness, relevance, and acceptability of each WHO-CST group session.

*Home visit feedback from caregivers *The caregivers completed the home visit feedback form containing specific questions for evaluating the usefulness and acceptability of home visits during each home visit.

##### Preliminary efficacy

*Caregiver knowledge and skills *The caregiver knowledge and skills test was used to assess the caregivers’ abilities regarding the WHO-CST program materials. Caregivers were invited to rate 24 statements on a 5-point Likert scale ranging from 1 (I strongly disagree) to 5 (I strongly agree). Statements included, “My child has more opportunities to learn when we are focusing attention on the same toy or activity”. Caregivers were asked to indicate their confidence level (13 statements) on a 5-point Likert scale ranging from 1 (not confident) to 5 (very confident), with statements such as “I feel confident in using pictures to help my child follow a routine.” Caregivers were also asked to complete three scenario-based short answer questions. This instrument was used during the first home visit (before the WHO-CST program), post-session 4 (mid-WHO-CST program), and the third home visit.

*Quality of life *The general health questionnaire (GHQ-12)^24^ was used for quality of life assessment to understand the state of health of the caregivers throughout the intervention period. Caregivers were invited to rate 12 statements such as “I am feeling unhappy and depressed” on a 4-point Likert scale ranging from 0 (never) to 3 (often). This instrument was used during the first home visit (before the WHO-CST program), at post-session 4 (mid-WHO-CST program), and during the third home visit (after the WHO-CST program).

#### Data analysis for Phase 2

All interviews with the MTs were transcribed verbatim and summarized by the PDF and RA. All caregiver focus group recordings were transcribed verbatim, and two additional coders undertook pattern coding to summarize major themes in the focus group interviews^25^. The first coder was a trained RA majoring in psychology, and the second coder was a trained RA with a master’s degree. The first coder developed the coding scheme after she had read all the transcripts twice. The second coder coded the transcripts using the coding scheme developed by the first coder. The coders’ inter-rater reliabilities for the focus groups were 0.96 and 0.97. Five transcripts were read in full to get an overall sense of the data. Themes were established, informed in part by the focus of questions asked (for example, “expectations of the program” and “foreseen challenges”) and from what emerged in the data. A list of codes was identified and structured according to themes and sub-themes. This was applied to all transcripts, and quotes in Cantonese were selected and assigned to codes. Common themes were identified within the interview transcripts by two RAs. In reporting results, representative quotes are selected to illustrate findings.

For quantitative data, descriptive analysis was conducted on all measures. Repeated measure analyses were conducted on data measuring the change in mental health and confidence in skill application. A paired-sample t-test was used to measure the pre-mid-post changes in caregivers’ knowledge attainment and quality of life throughout the intervention. Since this was a sequential mixed-methods study, a triangulation of qualitative and quantitative data from multiple informants with a convergent and parallel design was adopted with a more rigorous research design during Phase 3 of the evaluation to inform the acceptability and feasibility of the adapted WHO-CST-HK for pilot testing.

### Ethical approval

The study was approved by HKU’s Human Research Ethics Committee (EA1901033). All data collection was conducted in accordance with HKU guidelines and regulations. All participants provided written informed consent to (1) participate voluntarily in the WHO-CST-HK and understood that they could withdraw anytime and consent for study participation on behalf of the children they took care of; (2) join the three home visits that would be videotaped with the presence of their children for research purposes only and consented on behalf of the children they took care of for videotaping; (3) participate in the online evaluation as part of the study; and (4) understood that the data collected during the study without identifiable information or image would be destroyed five years after the first publication of the study.

## Results

### Phase 1: Cultural adaptation of the WHO-CST-HK

Overall, all participants supported the implementation of the WHO-CST in Hong Kong. In general, all stakeholders welcomed the potential introduction, adaptation, and wide implementation of the WHO-CST in social services settings. All caregivers had experiences of participating in various types of parent training programs. Most participants felt the WHO-CST-HK would fill the service gap caused by the increasing service demand from families of children with potential ASD and DD. Nevertheless, some participants raised concerns about potential challenges and the need for enhancements during the program implementation process. Some suggestions were reported to the WHO-AS team and incorporated into the pre-pilot field testing for further evaluation.

#### Adaptation meetings with stakeholders

Stakeholders shared their perspectives on the feasibility of the program in Hong Kong. Four major overarching themes emerged after analysis: (1) sustainability and the local context; (2) training and supervision of MTs and facilitators; (3) adaptation and implementation plan; and (4) language and cultural considerations. Stakeholders provided insights into how challenges could be handled (see Table 3).

1. Sustainability and the local context
  Many stakeholders reported a growing number of social services for families of children with potential ASD, DD, or other disorders. They believed that the inclusive nature of the WHO-CST-HK would address a wide variety of HK families’ needs, especially those that could be engaged in the social services setting before receiving medical services that require certified diagnoses by mental health professionals. One stakeholder highlighted that teachers in kindergarten and primary schools would also benefit from learning and using the skills offered by the program. Stakeholders particularly supported the local sustainability of the program, stating that it could be integrated into pre-existing social services, especially through the 19 subsidized Parents/Relatives Resource Centers, five of which have been set up with specialized ethnic minority units that aim to provide community support for the parents and relatives/caregivers of persons with disabilities. With assistance from staff, caregivers learn how to take care of their family members with disabilities or with difficulties in upbringing, exchange experiences, and establish mutual support.
  There were concerns that some of the potential program deliverers may not be ready to shift their delivery of care from the tertiary sector to the primary sector, which may impact the program’s sustainability. In addition, there was concern that the home visit component might not be well-accepted by local families. It was mentioned that some families may be over-concerned about confidentiality and reluctant to, or refuse to, invite “strangers” to visit their homes for evaluation and the provision of services. However, some stakeholders reflected that in their experiences conducting parent training programs, home visits are important to build initial engagement between the interventionists and the caregivers. They suggested that the purpose of home visits should be explicitly stated at the outset to alleviate potential concerns.
2. Training and supervision of MTs and facilitators
  The inclusion of MTs and facilitators from a variety of backgrounds was generally supported. However, there were concerns about whether caregivers would accept facilitators without professional qualifications and clinical experiences. One stakeholder explained that local families preferred programs led by professionals and the task-shifting or task-sharing concept might not appeal to them. However, it was understood that it was a core principle of the WHO-CST. The difficulty of engaging caregivers in training due to their busy work schedules was also mentioned. Some stakeholders suggested that ongoing training and supervision for the MTs and facilitators, offered by WHO and AS, were needed to enhance the credibility of the WHO-CST. Various strategies were suggested based on the stakeholders’ experiences organizing parent training programs, such as reviewing synchronized video sessions and online training.
3. Adaptation and implementation plan
  Several seasoned stakeholders raised concerns regarding the need to limit the number of group sessions (e.g., from nine weekly sessions to six weekly sessions) and to shorten the duration of each group session (e.g., from 2 to 1 h) due to the caregivers’ busy schedules. However, a few experienced stakeholders, particularly parents’ representatives, supported following the original version of the WHO-CST program rather than bringing in preconceived ideas about the program (e.g., long schedule) without trying out the first round of actual implementation.
  Another important aspect of the program is to collect and analyze the data to examine the feasibility and acceptability of the locally adapted program. However, there were concerns about the future feasibility of ongoing research among service providers. One stakeholder indicated that social workers and other health care providers might not realize the importance of research. Hence, frequent communication with staff about the rationale for the research was suggested. Moreover, a few stakeholders anticipated difficulty in recruitment for the pilot stage. However, one stakeholder stated that recruiting subjects was not that difficult if the study was carefully monitored and explained to the caregivers in detail so that parents would, in those circumstances, be aware of the importance of commitment throughout the study.
4. Language and cultural considerations
  Most of the language considerations were limited to specific words, which may be problematic when materials are translated into Chinese. A member of the adaptation advisory group suggested that the “mood thermometer” was an abstract concept that may not be easily understood. The group suggested using emotional adjectives to make the concept more relatable. However, other stakeholders shared their successful experiences using the mood thermometer in the local context in other programs. Therefore, the item remained unchanged. Members of the adaptation team also found that certain content did not apply to the local context. Some stakeholders noted that the concept of family sins is inappropriate in Hong Kong culture. Deletion of such content was undertaken during the review of the materials. A few stakeholders suggested that the pictures in the booklets should be replaced by more local scenes and the names of the vignettes replaced by Chinese names. However, to retain the cultural inclusivity nature of the WHO-CST, the pictures and names were not changed in the adapted materials. In summary, only minor adaptations were included in the adapted materials which included agreements on the name of the programme and the proposed terms in Traditional Chinese (e.g., Joint Engagement), localized daily examples (e.g., irrelevant cause to developmental delays like “Sins of Family” was not emphasized in Hong Kong cultures and was reordered on the list), and additional support to participants to join the sessions (e.g., babysitting services provided either by the Team members or NGOs).

**Table 3:** Themes and selected narratives of all three adaptation meetings with stakeholders in phase 1 (n = 34).

Themes	Selected narratives
(1) Sustainability and the local context	(For) child and adolescent psychiatric services, we have a severely long waiting time, hence there is an extreme demand for services Most caregivers are misinformed or uninformed about what is really happening in HK. Many caregivers are deprived of information…Let’s find a way to reach those who are in need. The very purpose of the (WHO-CST) programme is to discover how to reach out to people at a low cost, make it sustainable, and make it effective…so (let’s) make it inclusive Many caregivers in HK are in need. No matter whether their children are diagnosed with special needs or not. They need these caregiving and play skills
	To a certain extent, teachers also need these skills, as they may not be intensively trained in (the) application of behavioural management techniques or communication techniques with children
	We conduct the home visit again to know what they have learned from the group and how they apply it in their home settings.…So we understand that home visits are very important to understand the family and their needs. Some of my colleagues (who) conducted home visits will know, in a real living environment, what kinds of strategies they can apply or cannot apply
	The health care services in here have been skewed in secondary and tertiary care…the Hospital Authority is now shifting the services to a more community-based level, (focusing more) on maintenance care and preventive measures…It was a great challenge, (since) not all psychiatric nurses are ready to equip (themselves) to deliver services to primary health care. It is a new chapter for us to reach out and to collaborate with community partners. It is another set of competencies that they need to develop and to communicate
(2) Training and supervision of master trainers and facilitators	We welcome caregivers or nonprofessionals as facilitators. Many years before we ran our training programmes in caregiver resource centres, and we trained a group of caregivers. They went through the difficulties, and they want to be the volunteers to partner with other caregivers of children who are newly diagnosed with ASD. So, we provide some training for them, and they became the coaches for other caregivers…. It’s very meaningful as many years afterwards, these caregivers still keep contact with the coach, such as “yum cha” [dining together], and they still support each other in the community in the long run caregivers could also be the facilitators…Peer caregivers regularly come to our centres and make some “care calls” to other caregivers. We also think that facilitators who are the caregivers of children from different age groups are good because ASD is a lifelong condition. At different life stages, caregivers encounter different challenges, and they could share their experiences of what they have gone through, so that caregivers could get some mutual support
	In caregivers’ minds, they trust professionals. If the facilitators are not professionals, they may not trust the programme, (for example), ….it is difficult to engage caregivers for sustainable training due to their family and work engagements
	Ongoing training and sharing among our staff are very important because during the process when we hold different groups, we may come across different types of difficulties or caregivers’ issues. (Hence) we continue with in-service training; we videotape some sessions when running groups. New staff and programme facilitators can watch the videos to learn to facilitate the skills that are more appropriate for our caregivers We may have difficulty in officially releasing staff members to attend the five-day training. It may help if the training can be delivered in another kind of format, like e-training or online training
(3) Adaptation and implementation plan	Let us not think about efficiency version or whatever…let’s just do the full version because that’s what it is basically designed for…Adaptation, to me, is to make the programme more efficient, try to connect with people more efficiently and meaningfully. Other than that, we should not make it more compromised. Let us find out and have it implemented in full and see… Just go straight away, to see how it (the WHO-CST programme) implements with caregivers as it also depends on the facilitators, how they (caregivers) interpret and localize it into daily life situations. Adaptation and translation are an ongoing process, with feedback not just from the caregivers, but also from the facilitators and all stakeholders
	Sometimes it is difficult for us to have a randomized controlled trial (RCT) study because most of the social workers and frontline workers do the frontline work with the caregivers only. But for the research study we must commit to lots of things, some administrative work, and some preparatory work…not really related to direct service. So, we must communicate with our staff and colleagues a lot first…we must educate our staff that it is necessary to do research. Sometimes we may forget about the study as we focus on the service
	Ongoing monitoring is very important…in the process it is also important to let our target caregivers know what we are doing because sometimes it is easy for them to drop out. Sometimes their child is sick, or other things. They may have to withdraw, or they may have to skip some sessions. So, at the start, we tell the caregivers what we are doing and there is research behind it…I think we must let the caregivers know the importance of commitment
	Ongoing monitoring is very important…in the process it is also important to let our target caregivers know what we are doing because sometimes it is easy for them to drop out. Sometimes their child is sick, or other things. They may have to withdraw, or they may have to skip some sessions. So, at the start, we tell the caregivers what we are doing and there is research behind it…I think we must let the caregivers know the importance of commitment

### Needs assessment focus groups with caregivers

The 15 caregivers with service experiences shared their perspectives on the implementation process, covering the following six themes: (1) directions of implementation and sustainability; (2) inclusion and exclusion criteria for target participants; (3) screening and recruitment procedures; (4) program content; (5) attributes of MTs and facilitators, and (6) assessment method for impact measurement—videotaping. Furthermore, several suggestions for improving the acceptability and sustainability of the program were provided for the Team’s consideration (see Table 4).

1. Directions of implementation and sustainability
  Most participants (n = 12) highlighted the importance of establishing clear goals and applicability to real-life practice when promoting the program. Caregivers valued programs focusing on strategies they could use in daily practices rather than theory-based ones. Some participants (n = 7) inquired about the uniqueness of WHO-CST. They mentioned that there were already many parent-mediated programs and information they could obtain from various sources in Hong Kong. They questioned the need for an additional program to achieve similar results. One mother, who had lived in Canada since she was a teenager and had recently moved to HK, provided an alternative view. She stated that many families in HK with a child with ASD were not yet prepared to receive services. Hence, the WHO-CST, being more inclusive for children with potential delays, might be appealing to caregivers who were reluctant to receive a proper diagnosis for their children.
2. Inclusion and exclusion criteria for target participants
  The inclusion criterion of a child’s age range was well-accepted by most participants (n = 10). They particularly appreciated that the program extends beyond 8 years old, which is the usual cutoff point for most services.
3. Screening and recruitment procedures
  Most participants (n = 11) supported using the ten-question screen as a screening tool. They saw it as a tool that allows parents to report a child’s problem without having to exaggerate to get an opportunity to join a program. Participants also suggested recruiting eligible participants through kindergarten and maternal child health clinics (MCHCs) under the Department of Health as most caregivers and their children need to receive services from MCHCs, and children must attend kindergarten.
4. Program content
  Since the WHO-CST is a multi-component program, participants recommended more time for caregivers to understand the materials and practice in real-life situations. Half of the participants considered the weekly session design was “too rushed” for caregivers. Bi-weekly sessions were preferred. They also suggested that sessions be held at convenient locations (e.g., near their homes) during weekday mornings while the children were at school to fit the typical schedule of caregivers better. Many participants saw the benefits of having home visits as part of the program component. They saw it as an invaluable opportunity to build connection and trust with the interventionists (i.e., MTs and facilitators), as well as an opportunity for interventionists to better understand the unique difficulties that the caregivers were encountering and offer specific intervention strategies in the home setting. One mother from an extended family suggested that interventionists play a significant role in teaching other family members to assist in managing the child’s problem. However, some participants were concerned about the acceptability of home visits due to their small living spaces and possible objection from other family members. One mother was concerned that the Chinese term for home visits symbolized that the child might not behave well at school.
5. Attributes of MTs and facilitators
  Another concern some participants (n = 5) raised was the professionalism of MTs and facilitators. From the caregivers’ perspectives, those who had attended various training programs before expected MTs and facilitators to be skillful, knowledgeable, and down-to-earth. These caregivers did not want their children to be “tested” or “experimented on.”
6. Assessment method for impact measurement—videotaping
  Some participants thought that videotaping at home would make them uncomfortable because of privacy and confidentiality issues. Furthermore, participants asked questions such as: “what is the purpose of videotaping?”; “how would the videotapes be handled?”; and “will the video records be disclosed for publicity, public education, reviewing with other caregivers during group sessions, and researchers to do the analysis?” One mother raised another concern about whether videotaping could have an impact on the effect of the WHO-CST program on caregiver-child interactions, given that the behavior and emotions of children with ASD can fluctuate significantly.

**Table 4:** Themes and selected narratives of the needs assessment focus group with caregivers (n = 15) in phase 1.

Themes	Selected narratives
(1) Directions of implementation and sustainability	The goal (of the WHO-CST programme) must be made much clearer, what target participants are looking for, because sometimes I found after I participated in a programme, oh (I) joined the wrong course, a wrong one The useful stuff I will take it, but not just theoretical inputs, because after you listen to them (note: skills learned from the training programmes), during that moment you may understand, but as time goes by, you would forget. It’d great if that moment it is useful One NGO, (it costs) one hundred dollars only (note: course fee), but I still don’t want to join, as it’s not attractive and has no practical use…it’s better than nothing, but if (those programmes) are more target-orientated for each individual child’s situation that’d be more attractive
	ASD families have ASD characteristics, right at the beginning you need us to commit to so many tasks (note: home visits, video recordings). I believe that you just simply scared people away. Then that means you would have only those families who are opened-up, and even without your programme they could still access services, they are able to search for resources that could support them
	Every year the school provides similar stuff (note: programme or course) to us, then a single education talk will be fine, it’s just similar, so why attend? Even (we need to) attend nine sessions, so please don’t bother (me), so some caregivers may think in this way. Teaching caregiving skills…in fact, hey they’re just all the same I do not know why the WHO needs to organize this programme? There are so many skills training programmes, and there is so much (information) online, up to the point that we cannot digest so much. If (you teach me) ten skills and I could use one to two skills at home, that could save my life, or relieve me. I think that is more important than that three-month WHO programme
(2) Inclusion and exclusion criterion for target participants	Yes, caregivers of children in different age groups need these supporting programmes. These services should not be discontinued when the child is at primary school At the beginning (when the child) is aged 2 years, (he/she) would be alert to what the adults are doing, so it’s better to start (the programme) at the age of two. The golden period would be on or before aged 8. That’s why all the resources are cut at age 8 for the primary schools and you are required to seek (resources) yourself. They are thinking everything is fixed already. There’s no more about (the child) that can be changed, nothing can be changed. So, for me, if the training could last till aged 9 it’s better
(3) Screening and recruitment procedures	Why could I not find these courses at the very beginning? …Oh! Indeed, when we (note: participant and her husband) knew (the results), I think it was not that case, so we didn’t do anything, my husband and I thought it was impossible, we thought he does not have developmental delay, the assessment had problems…
	I think in MCHCs we need to have a pamphlet (about the WHO-CST programme) that means to alert the caregivers, so if you encounter your child’s situation, you could search for these programmes to offer help, but it is unnecessary to wait for the services provided by Child Assessment Centre, which means it would be a long waiting journey To make the kindergartens better aware (of the child’s problem), if some teachers have such alertness, and (they know) the child has such problems, they could tell the (caregivers) there is a programme (called WHO-CST programme) and suggest they participate in that, which could save time and resources
	I am so excited about what you’ve just mentioned (note: about the Ten Question Screen). I’d say please do not rely on those reports. Those reports are based on what the caregivers told (to the doctors) during the interview. Often caregivers would share what they perceived their child’s problems to be. Because of fighting for resources, (many caregivers) would rather seek for the reports, so if (you) want to serve the caregivers, the principle is to ask less. It’s not necessary to obtain so many child reports
(4) Programme content	Too rushed. I have not understood most of the materials and then the next session, the programme (sessions) are too packed, because every week we need to attend, (we have) to understand, to digest, and then to use them. It is very demanding Because for my child, it is not like an injection, it’s not like after the nine sessions that suddenly (my child) would know everything all in a sudden, it’s not like that, it takes time, or during practice if I encounter difficulties, I’d not know how to solve them
	The benefit (of arranging the programme) on a bi-weekly basis is it will not be too difficult, for traveling back and forth…otherwise I won’t have my own time, or time to rest. (I) won’t have time for the housework
	You really must visit to his or her home, possibly (you) may realize lots of issues, and (the interventionist) could directly point out the issue, and demonstrate to you at once, (for example), how difficult it could be to help the child to wear a pair of shorts, even though for helping the interventionists, I think they could understand It’d better to have some “tailor-made” time for us, that means (we could) raise questions, such as how to manage our child’s problems, for the home visits, if some professionals could provide one-to-one service, that’d be appropriate. Because there are many training programmes that could serve general situations, but they’re not specific enough… You come and understand what exactly the problems are currently facing each individual family, and then the trainer could know how to train the entire family as the ASD problem could be associated with the entire family unit
	Face-to-face (interviews) could allow the interventionist to get in touch with the entire family, teach the grandparent caregivers, or teach the father or the brother, so (home visits) are good, and the interventionist could observe the entire home environment
	You know the living space is small in HK, that means I may not (allow home visits), and for those living with grandparent caregivers, they may even refuse the home visits, and even three visits seem too much
	With the term “home visit” I could feel pressurized, as in HK, the only time when you need a home visit is because your child misbehaves at school. You’d immediately think of something bad (about your child)
(5) Qualities of master trainers and facilitators	Maybe for those so-called professionals who just received training from a lecture, (they) may not have much experience in managing a child, because studying and conducting real-life practice is different. I am expecting that at least they have experience in caring for these children, know how to play with them, and know how to communicate with them For those who just recently graduated and are just treating your child for the sake of doing an experiment, then please don’t. It’s not that I don’t want to spend the time. We don’t have much time to be spent (in this way). And I even don’t have time to tackle our child’s problem, so how come I would have time to spend for those so-called professionals
(6) Assessment method for impact measurement—videotaping	I am so afraid, oh my god if you really did the video recording, how would you handle the tapes? Because it’s just like taking off all your clothes. Let the others see what’s going on. Your home is so messy, or your home is that dirty. caregivers do mind this, would have feelings of discomfort If video recordings are made, I could not be that relaxed, and I’d considered the video records as taboo. There’ll be some requirements. (I) don’t feel very comfortable with that
	The child’s performance could fluctuate, because even if (the child) is at home, for that moment, whether you could record that situation it’s hard to say. You may not really be able to see their improvements, and I think (that improvement) it’s just by chance, so I think you may not see the effect
Suggestions for improvement	After I complete the program the service would be immediately cut off, so what shall I do? If it isn’t, we do need to have back-up, can approach the nurses or social workers. It could be apps, it could be very simple, just like these focus group interviews. (We) could have a very informal discussion, these could help the caregivers a lot… There is a platform for us to share, if I encounter this problem, if I forgot something, or anything that (I) could do better…indeed I found (this support) is important
	My husband still doesn’t accept (the child as having ASD). It has been three years that he doesn’t accept it. The caregiving strategies won’t be consistent with each other. There are lots of arguments throughout the day. An all-rounded (programme) would be better that serves couples, for one to two sessions that serve couples, it’d be great

#### Suggestions for improvement

Some participants proposed several strategies to improve the feasibility and acceptability of implementing the WHO-CST program. For example, they suggested conducting a briefing session for all the eligible participants of the WHO-CST program before the first round of home visits. This briefing session could address the need for home visits and video recording so that caregivers have a clearer idea of the implementation process. Another important issue concerned ongoing support after completing the program. Strategies such as using follow-up calls, apps, focus group interviews, and an online platform for the caregivers to share their caregiving experiences were proposed. Three participants raised poor couple relationships. These participants stated that their husbands found it difficult to accept that their child had ASD or DD. They suggested that the WHO-CST program content could address couples rather than individual caregivers.

### Phase 2: Pre- and post-intervention focus group interviews with caregivers and MTs and quantitative findings of the pre-pilot testing

Seven pre-intervention and five post-intervention focus groups with caregivers were held. The major themes included: (1) perceived benefits of the program, (2) useful elements in the program, (3) environmental and cultural barriers, and (4) major challenges and proposed solutions (see Table 5 for more details on the narratives).

**Table 5:** Themes and selected narratives pre- and post-intervention focus groups with caregivers in phase 2.

Time point	Themes	Selected narratives
Pre-intervention	1. Hope to better understand their child	So I want to communicate more with (my child), firstly for more communication, and secondly to engage in paired reading more often …teach him how he should express himself and how he should act
	2. Learn skills	Usually, after learning skills from other courses, they are hard to implement back at home. Now that you will come to my home, I am hoping that you could tell me how to better train my child in my own setting
	3. Manage inappropriate behaviors	Sometimes he doesn’t like to wash his hair, he can throw a big tantrum like crazy. I want to learn something about it
	4. Home visit barrier	I and my wife hold different opinions on the matter. That’s why sometimes you will see me accepting certain situations, but she doesn’t. Sometimes I think it is right, but she does not, something about order. We also have an elderly relative at home who may be uncooperative at times
	5. Unsupportive family members	Even if you asked him, he won’t come over
Post-intervention	1. Reduction in challenging situation	Initially we would fight over something constantly, but later in the programme, the fighting reduced. There is literally no more fighting over anything
	2. Learned skill	There is no third choice of toys set [the choice of NA], nothing to choose from
	3. Understand inappropriate behaviors	Now that I have taken the course, I understand my child’s behavior. I know what methods I could use to help him, now his (behavior) improved. My angle and stance changed

#### Pre-intervention focus group with caregivers

Before joining the program, caregivers expressed hopes for improving communication between themselves and their children, better understanding the needs of their children, and preparing their children for social situations. Some caregivers expected to learn skills that would better fit their own situation. Some caregivers hoped to understand and manage their child’s inappropriate behavior.

*So I want to communicate more with (my child), firstly for more communication, and secondly to engage in paired reading more often.*

*… teach him how he should express himself and how he should act.*

Some caregivers expected to learn skills that would better fit their situation.

*Usually, after learning skills from other courses, they are hard to implement back at home. Now that you will come to my home, I am hoping that you could tell me how to better train my child in my own setting.*

Some caregivers were hoping to understand and manage their child’s inappropriate behavior.

*Sometimes he doesn’t like to wash his hair; he can throw a big tantrum like crazy. I want to learn something about it.*

Many caregivers expressed hopes that home visits would help them implement the skills taught during sessions and help them choose the right skill to use. However, many came across the barrier of their other family members not supporting their decision to join the program.

*My wife and I hold different opinions on the matter. That’s why sometimes you will see me accepting certain situations, but she doesn’t. Sometimes I think it is right, but she does not, something about order. We also have an elderly relative at home who may be uncooperative at times.*

Many caregivers understood that the older generation may have a different caregiving method and were worried that it might clash with skills taught in CST. In addition, caregivers lacked the confidence to carry out the home practice. Caregivers expected the child to be uncooperative during practice and were not confident they could apply the learned skills.

#### Post-intervention focus group with caregivers

During the post-intervention focus group, five prominent themes emerged: (1) overall reduction in challenging situations, (2) improvement of caregiving skills, (3) management of own emotions as a caregiver and taking better care of themselves, (4) understanding and management of a child’s inappropriate behavior, and (5) home visits as a helpful element. Some caregivers reported observing a significant reduction in challenging situations. They learned to offer only two choices instead of three to reduce the possibility of bargaining. As a result, many felt that they had improved their caregiving skills. Caregivers also recognized the importance of managing their own emotions, particularly after the final session. They reported focusing more on themselves, which helped them improve their caregiving skills. Caregivers indicated that CST helped them understand their child’s inappropriate behavior from a different perspective. All caregivers also agreed that home visits were particularly helpful in enhancing caregiver skills. Many stated that they would like more time per visit, and some wanted to have a copy of their own video for revision and as an example for other family members. After participating in all the CST sessions, caregivers reported a significant reduction in challenging situations.

*Initially, we would fight over something constantly, but later in the program, the fighting reduced. There is literally no more fighting over anything.*

*There is no third choice for toys to play with during the practice, nothing more to choose by my child, and it looks boring.*

Caregivers learned to offer only two choices of toy sets, with no third choice for bargaining—the child must pick between the two. As a result, many felt that they had improved their caregiving skills. Caregivers also recognized the importance of managing their own emotions, particularly after the final session. They reported focusing more on themselves, which helped them improve their caregiving skills. Caregivers indicated that CST helped them understand their child’s inappropriate behavior from a different perspective:

*Now that I have taken the course, I understand my child’s behavior. I know what methods I could use to help him. Now his (behavior) improved, my angle and stance changed.*

All caregivers also agreed that home visits were particularly helpful in enhancing caregiver skills. Many stated that they would like more time per visit, and some wanted to have a copy of their own video for revision and as an example for other family members.

#### Pre-intervention interviews with MTs

All interviews were structured around discussing the relevance of the program with local families, foreseen difficulties, suggestions for improvement, sustainability of the program in HK, and drivers of their continuous contributions to the program. Five themes were identified: (1) caregivers’ needs and the program; (2) suggestions regarding the implementation of the program; (3) recruitment of caregivers; (4) families’ engagement/attendance; and (5) comments on training for service providers (see Table 6 for selected narratives).

1. Caregivers’ needs and the program
  Most MTs (n = 6) believed that the program could satisfy the demands of local caregivers and would be welcomed. The program provides a novel parenting method and could supplement and fine-tune caregivers’ parenting skills. The program could also fill the gap in relationship building within families, which was lacking in certain districts. While the content and objective may be highly relevant to local families, MTs suggested providing a clear objective with relevant topics to caregivers in each session to enhance their expectations and motivation. One MT was concerned about the effectiveness of learning through the story-based content. She suggested focusing on discussions and connecting the insights with caregivers’ daily life experiences. Another MT suggested allowing caregivers to bring their children into the session so caregivers could practice and receive feedback immediately. This would also ensure the children were cared for while the caregivers attended the session.
2. Suggestions regarding implementation of the program
  MTs found it challenging for caregivers to stay focused for the 3-h session and saw the need to adjust the session length and fine-tune the course content.
  One of the most discussed components was home visits. MTs believed that home visits would be welcomed once the relationship between the caregiver and the facilitator was established. Some MTs were concerned that three home visits would be difficult to execute due to limited staff availability and other work engagements. They expected they would have to use their annual leave and weekends to complete all the visits. One MT also suggested moving one of the home visits to a service center to reduce the provider’s burden. If this was accepted, they were willing to increase the number of home visits.
  MTs also raised concerns about the video recording component. They explained that some parents were hesitant to disclose their home environment to others; therefore, a clear purpose for video recording was needed to explain to caregivers. Another MT believed that caregivers are generally comfortable with video recording as long as the rationale and expected outcomes are clearly defined. Confidentiality should also be assured to increase acceptance.
3. Recruitment of caregivers
  As the program was promoted with a set of research criteria, some MTs were concerned that caregivers might not be honest while reporting their eligibility to secure a seat in the program. In particular, one MT found it hard to recruit in her center as most of the children they serve could speak complete sentences and thus were ineligible. One MT suggested putting caregivers in similar situations into the same class to facilitate the learning atmosphere and consistent attendance.
  In addition, MTs suggested promoting the program through social media, effective mediums (such as Maternal and Child Health Centers), and caregivers’ word of mouth. Attractive components such as brand names like WHO and HKU, an effective marketing plan, and certification upon training completion should be included to enhance promotion efforts.
4. Families’ engagement/attendance
  MTs reported that local caregivers often have to care for more than one child, and thus it would be challenging for them to attend every week. Caregivers also lack support from their partners, which may hinder their participation. It was suggested that motivation and outcomes should be clearly justified to enhance attendance. It was also suggested that explaining the course load and approximate time required to attend the whole program to parents before joining the program could be helpful in encouraging attendance. Another MT also highlighted the importance of accounting for common caregiver agendas and convenient locations to best fit the caregivers’ preferences.
5. Comments on training for service providers
  All the MTs appreciated receiving training directly from WHO trainers. In particular, they could learn from the live demonstrations and receive immediate feedback in practice sessions. The training for trainers deepened their understanding of the essence of joint attention and other core concepts. One MT reported seeing the effects of CST on a child, which helped her gain confidence. It was suggested that more live demonstrations would prolong memory and enhance the training outcome. Conversely, how to effectively execute the concepts was lacking in the training. Another MT suggested teaching the rationales behind the concepts in practical ways during training.
  Some MTs (n = 3) found that the fidelity evaluation was challenging. They believed that the play techniques and experience required time to develop and suggested having clearer information on standards. Furthermore, they wanted to share their fidelity comments with each other to understand the passing criteria better. Another suggestion was that videos of successful demonstrations could be shared for reference to facilitate future facilitators in their process of training.
  To ensure facilitators’ quality in the long run, MTs believed that workers, such as school-based social workers, with deep knowledge and experience in developmentally delayed children would be suitable facilitators. Another MT mentioned that the facilitator’s acceptance of the child-parent relationship was also important. However, it is also possible to have paraprofessionals, adhering to WHO’s concept, as facilitators after the initial stage.

**Table 6:** Themes and selected narratives from pre- and post-intervention interviews with MTs in phase 2 (n = 10).

Time point	Themes	Selected narratives
Pre-intervention	1. Caregiver’s need and the program	[CST] is very different from the other services we usually see within our district, often times, caregivers felt like once their child attend training then that’s it, I (caregiver) don’t have to do anything at home. I think this [program’s] concept is very different Every [CST] session has its own focus, content which it wants to deliver through the stories, but [caregivers] have to be familiar with it first, or else it would sound really boring…how caregivers could retrieve insight from the story, instead I think it is more important for them to share their experience
	2. Suggestions towards implementation of the program	[if we tell the caregivers] we just want to bring the skills taught in the group to them, in relation to the toys they have and the home environment, we want them to truly be able to use the skills. They will be ok with it About video-recording, I will tell them, it is just a few cameras facing [the caregiver] and the child, we won’t see how many rooms you have, how many people are there, they will be fine afterwards
	3. Recruitment of caregivers	The major problem is that caregivers often hide things, that is how many services are they having, some are already receiving service but doesn’t report that, when we screen, we would tell them that the child has to have no diagnosis or they should have no parenting training experience, then they would not tell us the truth, or not the complete picture If, within the group, everybody has similar problems, or they share similar conversation topic, I think they will have a better chance of staying, or [make each session] a chance for them to share with people who understand them
	4. Families’ engagement/attendance	caregivers also have to see, the difference after attending the program, or change they have been developed, they would be more willing to engage
	5. Comments on training for service providers	Actually, is it possible to have a complete, or at least observe a person leading sessions so I could learn, that will be much more complete, it is also easier to understand the concept, currently everything is just piece by piece…sometimes it feels like “what do you want” Or they should do a demo, how is the process suppose to be done, how should we separate those 30 min, that is what do you do in the first 15 min, and then what after the 15 min, the main goal is what do you have to do, it would be nice to have more of those so we could better understand We will share our success with them [future facilitators], how to become a successful facilitator, I believe each of us [MT], after what we’ve been through, although frustrating, but I think it was a fruitful training. We also have something we could tell our facilitators, experience sharing is important…if we could pass the 90% fidelity, I think that video could be shared, and use it to train other facilitators so they don’t have to be as puzzled
Post-intervention	1. Fine tuning of program content	I think the content, the content of each session, is very repetitive. Since it was so repetitive, some of the [harder] ideas were not easily understood by caregivers caregivers had lots of inquiries when we taught about challenging behaviors, there were some solutions in the program but I feel like it isn’t enough to handle the inquiries, I feel like it is not able to handle a wide variety of problems When WHO trainers came, they’ve spent a lot of time on the theory and so we are very clear about the theory but they didn’t have [the opportunity to] demonstration skills, whether in video format or live, so we were not sure how to execute the skills
	2. Length of sessions	…we won’t talk about the stories only, sometimes if we are rushing, we might not talk about the stories and do role-plays instead, more beneficial 2.5 [hours] is too much…even if we have a helper to babysit the children in our Saturday classes, the helper is also very tired, everybody is tired, it would be best to keep under 2 h
	3. Importance of home visits	The time devoted to the program was long, but we found it worth the time, particularly the home visits [home visit] was a bit tiring, particularly the last ones in a day, but it isn’t a big problem
	4. Improvement for facilitator training and fidelity	They have their limitations and we were limited by time as well, you know [because of flight delay]. I think we’ve fully utilized the 4 days available, it’s just that the content could be more comprehensive I think we could show our recorded group session [to future facilitators] It is better if they let us know everything in advance, that is the criteria to be an MT

#### Post-intervention interviews with MTs

During the post-intervention interviews with all MTs, suggestions were provided in the areas of (1) fine-tuning program content; (2) length of sessions; (3) importance of home visits; and (4) improvement of facilitator training and fidelity.

1. Fine tuning program content
  Two MTs found the program content involved too many repetitions and lacked practical solutions for handling various situations. In addition, they believed that caregivers in Hong Kong were generally more educated and informed than caregivers in LIMCs. Since many parents had already been trained in some other programs, the content of this program was somewhat repetitive. They commented that pictorial materials in the caregivers’ booklet, such as the picture schedule and the thermometer, were especially welcomed by caregivers. The traffic light metaphor was effective in helping caregivers understand the content. One MT added video illustrations of the content within her group to aid learning. MTs suggested including more visual cards to assist participants in remembering the content.
  Some MTs reported that the material for Session 8 was helpful for caregivers, even though it was not directly related to caring for the children. One MT hoped that more self-care content could be added in each session in addition to the already present relaxation exercise. Another MT specifically mentioned that stories were depicting scenarios too lightly, giving the wrong impression that problems could be solved easily. Content on teaching a child to communicate was too simple for many caregivers as their children were already verbally competent. It was suggested that communication content tailored to higher-level skills should be included, and socially inappropriate speech should be addressed for caregivers in Hong Kong. Another option could be to offer two program levels to cater to different demands.
  Facilitator materials were dense, and some potential facilitators found the content hard to follow. It was suggested that facilitators would understand the materials better if elaborations and supplementary information came after core concepts and methods to apply the concepts.
2. Length of sessions
  Discussions and role-plays with caregivers were reported as particularly helpful for caregivers to understand the skills taught. Most MTs (n = 8) suggested that more time should be allocated to these two activities in each session. The 9-session design was acceptable as it is similar to other parenting programs. However, the session duration was considered quite long for some busy caregivers. One MT, whose venue provided childcare services for the group, reported that children “kept looking for their caregivers.” Half of the MTs believed 2 h was the optimal length per session.
3. Importance of home visits
  Most MTs found home visits helpful in understanding the caregiver-child interaction within the home environment, offering tailored advice, and increasing engagement during sessions. However, better planning and execution were needed. Many MTs found home visits tiring and demanding. They suggested that the format and requirements for home visits should be outlined to caregivers before joining the program so their expectations could be managed. Interventionists should also be reminded to set time boundaries to avoid having to visit during odd hours. Interventionists should also inquire about toys that the child was obsessed with before visits and avoid using them.
4. Improvement of facilitator training and fidelity

Some MTs found that the program had a lot of materials to go through, and time in training was insufficient. The fidelity assessment was also too challenging and lengthy for most MTs. It was suggested that WHO and the local adaptation teams clearly outline the assessment criteria at the beginning of training. The training focused on practice delivering the intervention, and more timely performance feedback was preferred. Sample videos with the WHO-CST team’s demonstrations were also suggested as a possible learning resource.

### Quantitative outcome measures

#### Feasibility

##### Attendance and attrition rates

*Dropouts *Two caregivers dropped out after the first home visit, before the first session, and one caregiver dropped out after Session 2. All reported work schedule conflicts as their reason for dropping out. Eight caregivers were unreachable before the end of the intervention. All other families remained engaged throughout.

*Attendance* The mean of attended group sessions is 7.55 (SD 1.61; range 2–9); 84.21% attended six or more sessions; 33.3% completed all nine sessions; 73.8% completed all three home visits.

*Adherence to home practice *The caregivers’ dairies revealed that 72.4% were able to complete home practices throughout the entire program. Before the second home visit, caregivers usually spent 15–30 min completing the practice (37.3%). At the end of the program, caregivers typically spent 10 min in home practice (35.5%). Practice frequency also showed a downward trend, from 3 to 4 times per week (31.3%) to 2 times per week (25.8%). Before the second home visit, 6.25% of caregivers found it difficult to remember to complete the home practice. By the end of the program, caregivers indicated that they did not feel confident practicing the strategies, had unexpected circumstances that interrupted their practice, and others kept interrupting their practice (6.5%). At both time points, a few caregivers found it difficult to find time for practice (9.5%). Overall, home practices were feasible for most caregivers (see Table 7).

**Table 7:** Factors impacting adherence to home practices.

	Mid-intervention (n = 32)		Post-intervention (n = 31)	
	N*	%	N*	%
**Contextual factor**				
Unexpected circumstance	6	18.75	3	9.68
Remembering to practice	4	12.5	0	0
Finding time to practice	5	15.63	2	6.45
Interruptions from others	1	3.13	2	6.45
Others don’t want me to practice	1	3.13	1	3.23
**Enactment factors**				
Did not know what to do	1	3.13	0	0
Did not understand the strategies	0	0	0	0
Did not feel confident	0	0	2	6.45
Strategies not appropriate	1	3.13	0	0
Difficulties engaging child	1	3.13	0	0

*Indicated that the statement to be 4 or above (4 = True, 5 = Very true).

##### Acceptability

*Post-session feedback by caregivers *Across the nine class sessions, session feedback indicated that caregivers found the sessions moderately useful and helpful (M 3.49, SD 0.11, range 3.76–3.95). Caregivers generally found the problem solving and self-care materials in Session 8 to be the most relatable (M 4.09, SD 0.82) and useful (M 4.13, SD 0.80). The most useful skill was tip 2 from session 1B, “Look and Listen—Catch your child being good and respond with praise” (57%) (see Table 8).

**Table 8:** Satisfaction survey of each session.

Session	n	Relatedness	Min.–Max	Usefulness	Min.–Max	Helpfulness	Min.–Max	Overall mean ± S.D
1A	37	4.08 ± 0.759	3.00–5.00	4.00 ± 0.624	3.00–5.00	3.97 ± 0.645	3.00–5.00	3.91 ± 0.600
1B	40	4.05 ± 0.677	3.00–5.00	4.00 ± 0.641	3.00–5.00	4.00 ± 0.599	3.00–5.00	3.90 ± 0.574
2	39	4.00 ± 0.761	3.00–5.00	4.00 ± 0.649	3.00–5.00	4.03 ± 0.584	3.00–5.00	3.91 ± 0.569
3	34	4.00 ± 0.779	3.00–5.00	4.12 ± 0.640	3.00–5.00	3.97 ± 0.577	3.00–5.00	3.85 ± 0.492
4	35	3.77 ± 0.770	3.00–5.00	3.94 ± 0.591	3.00–5.00	3.861 ± 0.601	3.00–5.00	3.76 ± 0.514
5	35	4.03 ± 0.857	2.00–5.00	4.11 ± 0.631	3.00–5.00	4.00 ± 0.642	3.00–5.00	3.89 ± 0.643
6	33	3.91 ± 0.765	3.00–5.00	4.00 ± 0.750	3.00–5.00	3.97 ± 0.637	3.00–5.00	3.82 ± 0.577
7	29	3.86 ± 0.789	3.00–5.00	3.97 ± 0.731	3.00–5.00	3.90 ± 0.673	3.00–5.00	3.79 ± 0.616
8	32	4.09 ± 0.818	3.00–5.00	4.13 ± 0.793	3.00–5.00	4.00 ± 0.718	3.00–5.00	3.95 ± 0.682

*Post-home visit feedback by caregivers *All home visits were considered quite useful and helpful (M = 4.317, SD 0.043, range 4.27–4.35). Video recording was also acceptable across all home visits (average mean 3.95, SD 0.566, range 3.89–4.00) (see Table 9).

**Table 9:** Satisfaction survey of home visits (HV) and video recordings.

	N	Min	Max	Mean	SD
HV (Pre)	40	3.00	5.00	4.33	0.572
HV (Mid)	37	3.00	5.00	4.27	0.693
HV (Post)	31	3.00	5.00	4.35	0.608
Video (Pre)	40	3.00	5.00	3.98	0.620
Video (Mid)	37	3.00	5.00	3.89	0.614
Video (Post)	31	3.00	5.00	4.00	0.730

##### Preliminary effectiveness

*Knowledge, confidence, and GHQ-12 *The knowledge score showed a positive trend in the mean score throughout the pre-pilot. The confidence and GHQ-12 scores fluctuated but maintained an upward trend within the range difference of 0.17 and 0.49, respectively (see Table 10). For most of the chosen evaluation scales, statistically significant differences were found in the caregivers’ mental health and confidence in caregiving.

**Table 10:** The descriptive statistics of the scores of the Knowledge and Skill Questionnaire, and General Health Questionnaire-12.

	N	Min	Max	Mean	SD
Knowledge (Pre)	38	4.00	55.00	26.24	11.66
Knowledge (Mid)	32	8.00	53.00	29.59	9.65
Knowledge (Post)	32	0.00	59.00	30.22	13.71
Confidence (Pre)	40	2.50	4.00	3.55	0.287
Confidence (Mid)	35	0.00	4.10	3.31	0.864
Confidence (Post)	33	0.00	4.80	3.38	0.930
GHQ-12 (Pre)	42	0.90	3.20	2.63	0.356
GHQ-12 (Mid)	37	0.00	3.00	2.38	0.864
GHQ-12 (Post)	38	0.00	3.00	2.14	1.05

## Discussion

This study examined the acceptability and feasibility of the WHO-CST-HK adapted for use in social services settings in the local context. This paper reports the triangulated findings collected from multiple informants in the first and second phases of the sequential mixed-methods evaluation study. Their experiences and perspectives of the WHO-CST and recommendations for improvement led to modification of the adapted version of WHO-CST to be further pilot tested in Phase 3 of the study^26^. Several relevant local and international recommendations have been generated from this study to enhance the sustainability of the WHO-CST as an important parent-mediated program for children with potential ASD or DD as part of the world movement for the mental health of children and adolescents and their caregivers^27,28^. The following discussion includes the important lessons and recommendations for improvement of the WHO-CST at the macro, meso, and micro levels to enhance the feasibility and sustainability of the program in HICs.

Overall, the program was accepted and supported by all involved individuals. Even though the WHO-CST program is a complex intervention with multiple components, it is feasible to scale up for further evaluation in the real community settings in Hong Kong. From a macro perspective, Hong Kong can integrate the program into the existing local social services system infrastructure. It is noteworthy that Hong Kong is not averse to adopting mental health promotion and intervention programs developed overseas. However, many overseas programs have been directly “translated” without much cultural adaptation for the local service users. The relatively large scale and rigor applied to the adaptation of the WHO-CST under the guidance of WHO and AS has introduced an invaluable experience to the local stakeholders, especially those from the funding agency and social services. The stakeholders are now well informed about the procedures, delicacy, and complexity associated with the adaptation of foreign programs introduced and implemented in cultures that may deviate from the program developers’ cultures.

If the WHO-CST-HK is scaled up, it will be worthwhile to consider that it can be delivered also in non-social service settings such as child services, foster care, preschools, nurseries, and kindergartens. It is important to note that many countries have adapted, and pilot tested the WHO-CST but have yet to enter the scale-up phase. More information about the implementation process and the contextual and cultural factors contributing to the scaling up process will further enhance the feasibility and acceptability of the WHO-CST as a worldwide, evidence-based, and endorsed parent-mediated program for families of children with suspected ASD and DD.

Incorporating routine home visits as part of an intervention by trained non-specialists has raised the most concerns among the local stakeholders and caregivers because of cost and privacy issues, respectively. Like previous studies^14–18,29,30^, once caregivers and program implementers experienced the home visitations, all of them praised the advantages since more individualized and tailor-made strategies could be generated from the home visits. The methodology for capturing data for evaluation during home visits, and videotaping, which was initially viewed as problematic, also received support and resulted in requests for the video for the caregivers’ own reference.

Task-sharing by non-specialists with little or no prior formal training or background in mental health care to provide brief interventions has been regarded as one of the best evidence-based practices mediated by WHO to deal with service gaps for mental health issues in LIMCs. Increased attention has focused on various approaches needed for effective implementation of service delivery by non-specialists in HICs because how task-sharing is implemented in less resourceful care systems does not necessarily apply in resourceful systems. Task-sharing in HICs would work best in a balanced and collaborative approach where non-specialists with robust training and supervision can work closely with the supervisors and other specialists in the system to “share” the heavy workload of the specialists throughout the broad spectrum of mental health care^31,32^. As Hong Kong is one of the few high-income locations to have adapted the WHO-CST, our experiences seem to be in line with Raviola et al.^31^ observation that it is better to prioritize the facilitator training and certification for professionals in the system as an additional qualification that allows them to provide specialized parent-mediated programs for children with potential delays. It is believed that the WHO-CST-HK can be more sustainable than training busy non-specialists who are often not in the system and are expected for long-term commitment as volunteers. More implementation process research is needed, especially about task-sharing in affluent places like Hong Kong and Italy.

As previously mentioned, at the meso level, the whole or partial WHO-CST-HK can be delivered by staff (e.g., teachers and childcare service providers for children with social and communication issues) in non-social services settings. This can be seen as part of a shared responsibility for the balanced and collaborative care for families of children with difficulties. This idea is believed to work effectively in places like HK, where access to information and communication technologies within a good digital contextual environment exists. The non-specialists with digital technologies and skills can be effectively supported by their direct supervisors in collaboration with specialists in the care system and empowered by improving access to training and skill building opportunities, supporting clinical care and decision making, and facilitating data collection and monitoring for quality assurance. At least two essential factors can facilitate the actualization of this shared responsibility movement: coordination by a committed government to improve mental health care and the sustainability of training and supervision of non-specialists.

Some local stakeholders raised significant concerns about these two aspects during the adaptation process. In particular, the MTs reported that the fidelity training delivered by WHO and AS is a unique experience that enhances the quality of service for caregivers. However, the process from completing the fidelity assessment to receiving the performance report and accreditation as a qualified MT was too lengthy for this three-year, territory-wide project. Given that the chief trainers from the WHO and AS are responsible for the training and fidelity assessment at all CST sites around the world, it is recommended that a regional trainee system supervised by recognized local MTs can be adopted to speed up the process of MT and facilitator training for larger-scale implementation of the CST program. The regional trainee system may also offer regionally relevant materials or information in addition to the original training.

At the micro level, most caregivers involved in the process found the adapted program’s content and delivery model acceptable and in line with their expectations. Some aspects of the program required minor adjustments or special consideration during the adaptation process. First, MTs reported that certain course content might not be suitable for the local population. In particular, the content on training a child’s language skills was not suitable because most caregivers had children who had already attained fluency in language production. This phenomenon is probably related to the strong focus on academic success and the importance of language in schooling among all caregivers in HK. The caregivers tend to see mastery in speaking and communication as prerequisites for academic success. Hence, caregivers in Hong Kong generally seek as much professional help as possible to support their children in acquiring language skills so that some level of academic achievement can be attained. However, as the original WHO-CST was developed and targeted at children who were minimally verbal^20,21^, the language section in the CST materials was less relevant to local needs.

Suggestions for improvements by some caregivers include enhancing the support through proactive engagement with busy working fathers and other elderly family members who see play as a less important aspect of improving children’s learning. In addition, a clinical psychologist with a South Asia background raised the issue of the challenges faced by non-Chinese citizens in Hong Kong, which has become even more challenging in the past two decades with more emphasis on using Chinese rather than English in daily activities. Hopefully, the recently launched WHO-CST e-learning platform will provide some short-term solutions to address these language challenges. For the longer term, more thoughtful planning to create a more diversified, inclusive, and equitable care system in Hong Kong and elsewhere is urgently needed.

It was encouraging to learn that most caregivers also observed the positive impact of WHO-CST in their daily lives in HK and other countries^16,17^. During the focus group interviews, caregivers explained how they used the specific CST skills to respond to their situations. Consistent with the literature, home practice sessions and discussion about caregivers’ experiences are effective methods for ensuring caregivers understand and use the skills taught^33^. However, programs of a similar nature are usually conducted in a one-on-one format, and caregivers in those programs lack the opportunity to develop a sense of peer support^34^. In the WHO-CST, caregivers and facilitators can work together and generate suggestions based on their personal experiences. This resulted in caregivers observing a significant reduction in challenging situations in daily activities such as dining and teeth brushing.

### Limitations

This study has several limitations. First, although the original WHO-CST was developed to address social communication and behavioral difficulties across a range of DDs, our program is part of a larger territory-wide program for children with ASD. Therefore, our study sample was skewed towards children diagnosed with ASD. Future research may target families of children with other forms of DD. Second, as many caregivers in our program have already sought help for their children with suspected ASD or delay issues, the unique impact of the WHO-CST-HK must be interpreted with caution. Third, it is unclear whether the study outcomes measured immediately after the intervention will be maintained over time. Fourth, although social desirability bias in qualitative interviews was minimized by having independent research individuals who were not involved in the program implementation process conduct the data collection that aimed to encourage caregivers to give their critical feedback about the adapted program, socially desirable answers cannot be ruled out. Fifth, many of the caregivers involved in the study were connected with the NGOs, so enhancing acceptance of the WHO-CST by caregivers who are yet to be connected with the service systems in HK should be explored in future implementation studies.

### Conclusion

Although the WHO-CST was originally developed for LIMCs, it is clearly appropriate to have the program in a scaled-up scope in Hong Kong. As caregivers of children with ASD and DD continue to rely on the public system for services and suffer from lengthy waiting times, the WHO-CST-HK has significant potential to support these families due to its task-sharing nature. The qualitative feedback from the pre-piloted WHO-CST-HK is positive, and the next step for the research team is to conduct a randomized controlled trial with sufficient sample size. The program could then be sustained with the support of the WHO, AS, local NGOs, caregivers, MTs, and facilitators in HK and for wider dissemination in other Chinese societies.

## Supplementary Information

Supplementary Information.

## Data Availability

The datasets generated during and/or analyzed during the current study are available from the corresponding author on reasonable request.
