# Supplementary material for: Adapting and pretesting the World Health Organization’s Caregiver Skills Training Program for children with autism and developmental disorders or delays in Hong Kong
Source: Sci Rep. 2022 Oct 8;12:16932. doi: 10.1038/s41598-022-21343-9 (PMC9547914; doi:10.1038/s41598-022-21343-9)
Supplement: Supplementary file 1 — Supplementary Information. [file 41598_2022_21343_MOESM1_ESM.docx]

Supplementary Table 1.1. Interview guide for needs assessment focus groups interviews in phase 1

| (1) | Could you share your experiences for recruitment and preferred settings for implementation? |
| --- | --- |
| (2) | Instead of using medical diagnoses as inclusion criteria for our programme, we are planning to use the Ten Questions Screen to screen and identify eligible caregivers, which may increase the chance for caregivers to participate in the CST programme. What do you think? |
| (3) | Caregivers attending the programme will be asked to practice strategies with role play and then to use these strategies at home with their own children. Will there be challenges to caregivers like yourself? |
| (4) | Caregivers participating in the CST programme will be asked to attend nine weekly group sessions and three home visits. Do you foresee any barriers with that? |
| (5) | What do you expect the attributes of competent master trainers and facilitators to be? |
| (6) | Do you have any suggestions for what to be aware of in implementing the CST programme? |

Supplementary Table 1.2. Interview guide for pre-intervention focus groups with caregivers in phase 2

| 1. | Do you find the CST goals and content relevant to your needs? Do you think it will be useful to caregivers like you? |
| --- | --- |
| 2. | Would you have liked the programme to address something else? Is there any other important message that should be conveyed to caregivers of children with developmental delays or developmental disorders? |
| 3. | Do you foresee any risk that the programme content contradicts important values of your family or community? |
| 4. | The programme aims to teach caregivers strategies to…. Caregivers attending the programme will be asked to practice the strategies with role plays and then at home with their own children.  In your view learning these strategies will be useful to caregivers like you? |
| 5. | Do you foresee any challenge with implementing these strategies at home with your child? |
| 6. | Caregivers taking part in the programme will be asked to attend 8/9 group sessions on a – basis, of the duration of…. The facilitators will also conduct three home visits… Caregivers will be asked to interact with their children in play for 10 minutes and, if willing, these play sessions will be videorecorded…  Do you think caregivers with children with developmental delay/disorders in your community will be able to attend such programme? Do you foresee any barrier with attending group sessions or being available for home visits? |
| 7. | Do you have any suggestion to improve caregivers’ attendance? |
| 8. | [Ask for any additional suggestion and thank participants] |

Supplementary Table 1.3. Interview guide for post-intervention focus groups with caregivers in phase 2

| 1. | After participating the WHO CST Programme, do you find the content is useful to caregivers like you? |
| --- | --- |
| 2. | After participating the WHO CST Programme, do you find it better to deal with the problems of children with developmental delays or disorders? |
| 3. | After participating the WHO CST Programme, do you find it is useful to reduce the stress of caregivers like you? |
| 4. | After participating the WHO CST Programme, do you find any improvement of children in language, emotion, social or repetitive behavior? |
| 5. | The CST programme aims to teach caregivers strategies in taking care of children who had been diagnosed or suspected to have developmental delays or disorders. Caregivers attending the program will be asked to practice strategies with role plays and then at home with their own children. Did you foresee any challenge with implementing these strategies at home with your child? How did you deal with it? |
| 6. | What factors affected you to implement these strategies at home with your children? |
| 7. | You have been taking part in 9 group sessions and 3 home visits, and also video-recorded during it. Do you face any difficulties? How do you solve it? |
| 8. | Do you have any suggestion to improve caregivers’ attendance? |
| 9. | What do you think was the most useful part of the WHO CST Programme? |
| 10. | How to further improve the WHO CST Programme? |
| 11. | Will you recommend other to participate the WHO CST Programme? |

Supplementary Table 1.4. Interview guide of interview with MTs in phase 2

| 1. | Introduce the topic of discussion. Ask about positive experiences. |
| --- | --- |
| 2. | Ask about negative experiences. |
| 3. | Ask about acceptability of the programme content |
| 4. | Ask about suggestions to make the materials more useful to caregivers and families. |
| 5. | Prompt for additional suggestion for change in the programme materials. |
| 6. | Ask about perceived impact of the programme on families. |
| 7. | Ask about barriers to implementation. |
| 8. | Ask about suggestions to improve strategies for training and supervision. |
| 9. | Ask about the usefulness of home visits. |
| 10. | Ask for any additional comments and thank participants. |
